# CAR T-Cell Therapy in Children with Solid Tumors

**DOI:** 10.3390/jcm12062326

**Published:** 2023-03-16

**Authors:** Marika Kulczycka, Kamila Derlatka, Justyna Tasior, Monika Lejman, Joanna Zawitkowska

**Affiliations:** 1 Student’s Scientific Association, Department of Pediatric Hematology, Oncology and Transplantation, Medical University of Lublin, Gębali 6, 20-093 Lublin, Poland; 2 Independent Laboratory of Genetic Diagnostics, Medical University of Lublin, Gębali 6, 20-093 Lublin, Poland; 3 Department of Pediatric Hematology, Oncology and Transplantology, Medical University of Lublin, Gębali 6, 20-093 Lublin, Poland

**Keywords:** adoptive cell therapy, CAR T-cells, solid tumors

## Abstract

The limited efficacy of traditional cancer treatments, including chemotherapy, radiotherapy, and surgery, emphasize the significance of employing innovative methods. CAR (Chimeric Antigen Receptor) T-cell therapy remains the most revolutionizing treatment of pediatric hematological malignancies and solid tumors. Patient’s own lymphocytes are modified ex-vivo using gene transfer techniques and programmed to recognize and destroy specific tumor cells regardless of MHC receptor, which probably makes CAR-T the most personalized therapy for the patient. With continued refinement and optimization, CAR-T cell therapy has the potential to significantly improve outcomes and quality of life for children with limited treatment options. It has shown remarkable success in treating hematological malignancies, such as acute lymphoblastic leukemia (ALL) and non-Hodgkin lymphoma (NHL). However, its effectiveness in treating solid tumors is still being investigated and remains an area of active research. In this review we focus on solid tumors and explain the concept of CAR modified T cells, and discuss some novel CAR designs that are being considered to enhance the safety of CAR T-cell therapy in under-mentioned cancers. Furthermore, we summarize the most crucial recent reports concerning the solid tumors treatment in children. In the end we provide a short summary of many challenges that limit the therapeutic efficacy of CAR-T in solid tumors, such as antigen escape, immunosuppressive microenvironment, poor trafficking, and tumor infiltration, on-target off-tumor effects and general toxicity.

## 1. Introduction

**Figure 1:**
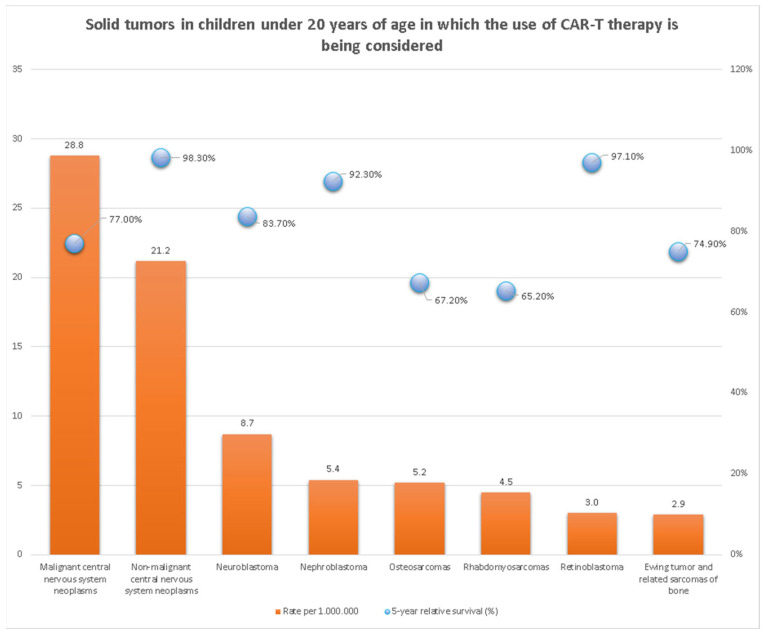
Solid tumors in children under 20 years of age in which the use of CAR T-cell is being considered [6].

Cancer is a significant cause of death among young people in the United States. For children up to the age of 14 it takes second place after car accidents, while in adolescents aged between 15 and 19 years cancer is the fourth most common cause of death [1]. Pediatric neoplasms are a heterogeneous group, which can be divided into hematological malignancies, central nervous system tumors, and extracranial solid tumors [2]. There are three predominant curative options for children suffering from solid tumors; surgery, radiotherapy, and chemotherapy. Surgical operations are useful, when a tumor is localized, small, and non-metastatic. There is a risk of recurrent malignancy in the case of incomplete resection. Chemotherapy and radiotherapy, despite their effectiveness, have a destructive effect on healthy tissues and may induce severe side effects [3]. Therefore, new therapeutic approaches such as CAR-T cells immunotherapy are being implemented. It has achieved significant success in the treatment of pediatric hematological malignancies [4,5], however, the data on solid tumors is still limited. This field is on the verge of revolutionizing cancer treatment and its prosperity is dependent on a number of restrictions, effectiveness, and safety, which seems particularly relevant for the purposes of pediatric oncology where the goal is to improve outcomes while minimizing toxicities and long-term side effects. As more clinical trials are conducted and experience with the treatment grows, researchers and clinicians will continue to refine and optimize CAR-T therapy to improve its effectiveness and safety. The statistics on the quantity and 5-year survival rate of pediatric solid tumors being considered for treatment with CAR-T cells are presented in Figure 1. For this reason, our study aims to gather and review the latest reports on the application of CAR-T cells in the treatment of solid tumors among children.

## 2. CAR Structure

The chimeric antigen receptor (CAR) is a bioengineered molecule which, combined with a T cell, can target the required antigen with a high specificity. CAR has the ability to recognize the tumor’s antigen without engaging the major histocompatibility complex (MHC), which prevents tumor immune escape during the immunotherapy. The CAR molecule consists of: the extracellular domain, the transmembrane domain, and the intracellular signaling domain [7,8,9].

During the last 30 years five CAR generations have been created. The first-generation CAR (1G CAR) contained only the extracellular domain with scFv and a single CD3ζ intracellular domain without any costimulatory molecules. By adding a costimulatory domain [e.g., CD28, CD134 (OX-40), or CD137 (4-1BB)] to the intracellular domain, researchers made a second generation of CAR (2G CAR), which resulted in increased in vivo lifespan of CAR T-cells. The third generation CAR (3G CAR) based on the 2G, differs only by an extra costimulatory molecule of CD134 or CD137. The fourth generation CAR (4G CAR) is also based on the 2G, yet it additionally expresses transgenic proteins such as interleukin 12 (IL-12) after the CAR activation [7,10].

**Figure 2:**
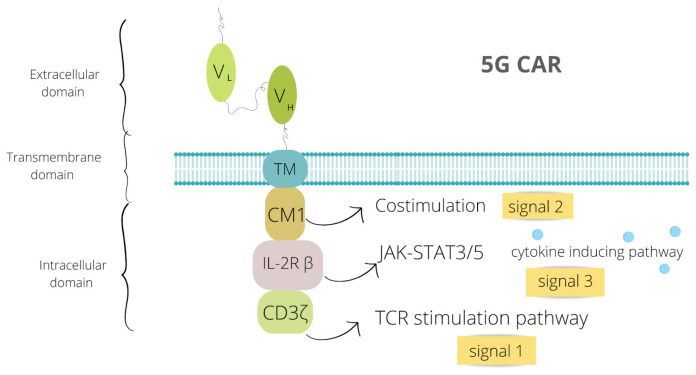
Structure of fifth generation of chimeric antigen receptor (5G CAR), also known as a next generation CAR. VL the light chain variable region, VH the heavy chain variable region, CM1 costimulatory molecule 1, TCR T-cell receptor. Image created with Canva Pro https://www.canva.com/pro/ (accessed on 27 January 2023).

The fifth generation, also known as the next generation, is now under research. The basis of its construct is a 2G CAR, yet it additionally contains a truncated cytoplasmic IL-2 receptor β-chain domain with a binding site for the transcription factor STAT3. This structure allows to react simultaneously and induce three stimulation pathways: TCR through the CD3ζ domain, co-stimulatory through the CD28 domain, and cytokine through JAK-STAT3/5. Thus, providing all these signals causes full T cell activation and proliferation [7]. The structure of fifth generation CAR is presented in Figure 2.

## 3. Pediatric Solid Tumors—Preclinical and Clinical Reports

### 3.1. Brain Tumors

The heterogenous group of childhood brain tumors divide into: (i) embryonal tumors—medulloblastoma, CNS PNET tumors and atypical teratoid/rhabdoid tumor (AT/RTs), (ii) gliomas—low-grade gliomas (pilocytic astrocytoma), brainstem gliomas (DMG/DIPG), non-brainstem high-grade gliomas and ependymoma, (iii) craniopharyngiomas, (iiii) pineal region tumors [11]. Different possible CAR-T targets such as interleukin-13 receptor alpha 2 (IL-13α2), human epidermal growth factor receptor 2 (HER-2), erythropoietin-producing human hepatocellular carcinoma A2 receptor (EphA2), disialoganglioside GD2 (GD-2) and B7-H3 are considered in pediatric cancers [12]. Recently, the latter two antigens have been assessed as particularly useful in pediatric high-grade-gliomas (pHGG). After analyzing patient-derived orthotopic xenografts (PDOX) the hierarchy of antigen pediatric brain tumor receptors has been established. GD2 and B7-H3 maintain the highest expression in pHGG as compared to IL-13Rα2, HER2, and EphA2 characteristic for adults [13]. Another study identified three cell surface targets, EPHA2, HER2, and IL13Rα2, that are expressed on medulloblastoma and ependymoma. Moreover, it confirmed that intrathecal delivery of EPHA2, HER2, and IL13Rα2 CAR-T cells is an effective treatment for primary, metastatic, and recurrent group 3 medulloblastoma and PFA ependymoma in a mouse model [14]. The new findings reveal a p32 as a tumor-associated antigen (TAA) in gliomas and consider p32 CAR-T cell immunotherapy as antitumor and anti-angiogenic [15]. In other research has been shown that the protein GPC2 is highly differentially expressed across a variety of pediatric brain tumors, including malignant embryonal tumors and a subset of HGGs and DMGs. These data show that GPC2 can be targeted safely with local delivery of mRNA CAR T cells, creating the basis for the clinical translation of GPC2-directed immunotherapies for pediatric brain tumors [16]. Another interesting finding is also the therapeutic potential of targeting IGF1R/IR in combination with GD2-CAR T cells to enhance antitumor efficacy in DMG/DIPG and possibly other pHGGs. In particular, the dual IGF1R/IR inhibitor linsitinib increased tumor cell death and enhanced the antitumor activity of GD2-CAR-T cells, providing a strong indication for the translational development of IGF1R/IR inhibitors as adjuvants for GD2-CAR-T in the future [17]. The B7-H3 (known also as CD276), a transmembrane checkpoint protein overexpressed in several cancers including pediatric solid tumors is another target for brain tumors CAR-T therapy [18]. In malignant tissues B7-H3 inhibits tumor antigen-specific immune responses leading to a protumorigenic effect. Therefore, a lot of antibody-based strategies targeted in B7-H3 expressing cancer cells have been developed and its efficacy is dependent upon high surface target antigen density on tumor tissues [19,20]. The results of another study suggest that B7-H3 CAR T cells are highly active against atypical teratoid/rhabdoid tumors (ATRTs) and that intraventricular delivery compared with intravenous may be preferable for the treatment of these tumors. They also showed a significant capacity of CAR T cells to mediate immunological memory both in the brain and in the periphery but further investigations on human tissue are needed [21].

Proceeding to the clinical data, one of the latest comes from the open, ongoing, phase I, single-institution clinical trial BrainChild-03, delivering locoregional B7-H3 CARs to children and young adults with recurrent or refractory CNS tumors and DIPG. The researchers demonstrated the feasibility of fractional intracranial B7-H3 CAR-T cell dosing and showed a connection between cytokine level and proinflammatory local immune cell activation in the CSF [22]. There is a promising treatment option for young patients with diffuse midline glioma (DMG) H3K27M mutated including DIPG. Particularly those mutated in the spinal cord are highly malignant [23]. Research has reported that disialoganglioside GD2 may be used as a target of immunotherapy in those brain tumors [24]. The currently ongoing phase I clinical trial showed radiographic and clinical improvement in three of four participants after intravenous administration of GD2 CAR-T Cells. The intraventricular administration of a second dose of GD2-CAR T cells intensified these improvements in three of three patients treated [25]. Vitanza et al. evaluated the feasibility, safety, and tolerability of local infusions of HER2-specific CAR-T cells in children and young adults with relapsed or refractory CNS tumors. Patients received infusion through a CNS catheter into the tumor cavity or ventricular system. Initial results showed clinical and correlational evidence of local immune activation such as the high concentration of cytokines [26]. Previous reports directed scientists to above-mentioned clinical trials [27]. In the same hospital another clinical trial is carried out. Patients with recurrent or refractory pediatric CNS tumors are monitored after the infusion of locoregional EGFR806-specific CAR T Cell [28]. The last clinical study worth considering in pediatric solid tumors is an assessment of third generation GD2 CAR-T cell therapy with the suicide gene safety switch—inducible Caspase 9(T iC9-GD2-CAR). That investigation is in the recruitment stage for children from 6 months old and young adults [29].

### 3.2. Neuroblastoma

Despite excellent results in treatment of localized disease, in patients with high risk neuroblastoma long term survival prospects are limited. Additionally, this population suffers from many adverse effects associated with curation. The success of anti-GD2 therapy has demonstrated that targeted immunotherapy can effectively treat neuroblastoma. Therefore, CAR T-cell therapy in neuroblastoma was the first beyond other solid tumors to reach clinical trials [30].

#### 3.2.1. Disialogangloside- GD2

In one preclinical study, first generation anti-GD2 (anti-disialogangloside) CAR T- cells were generated to demonstrate their efficacy in GD2-positive neuroblastoma. Unfortunately, lack of the costimulatory domain resulted in decreasing of their functionality over time, showing that CD3ζ intracellular domain is not enough [31]. In one Phase I clinical study, Epstein Barr Virus-specific cytotoxic T lymphocytes (EBV-CTLs) with a first generation anti-GD2 CAR were tested in 19 EBV-positive patients with refractory and relapsed neuroblastoma. In this trial, 11 individuals were treated with CTLs and ATCs, which are also GD2 CAR, yet T cells are activated through the native TCR (GD2 CAR-ATC). The GD2-specified CAR vectors were made from 14G2a antibody. Every patient received an injection of equal amounts of CAR-CTLs and CAR-ATCs. Measuring the PCR signal from the vector associated with CAR- CTLs and the other associated with CAR-ATCs demonstrated that CAR-CTLs were detectable for 6 weeks, comparing it with 2 weeks durability of CAR-ATCs. From eight patients with evaluable tumors, half of them (4) responded with tumor necrosis or regression [32]. There is a report that followed up the longer clinical response and CAR–T-cell analyses from these patients and from eight other remaining individuals. These data showed that long-term persistence of CAR T-cells improves outcomes and longer time to progression (TTP) [33].

In another phase I clinical study, 11 patients with high risk persistent or relapsed neuroblastoma were treated with third-generation anti GD2 CAR T-cells. In the trial, every individual was treated with one of the three cohorts; 1 cohort received only CAR-T, 2 cohort was treated with CAR-T and chemotherapy, 3 cohort received CARTs with chemotherapy and a checkpoint blockade drug- a programmed death-1 (PD-1) inhibitor. The lymphodepletion activated by chemotherapy in cohort 1 and 2 expanded the level of CARTs. PD-1 inhibitor did not significantly affect expansion or persistence. None of the three cohort treatments indicated measurable antitumor response [34]. Subsequent Phase I clinical trial enrolled individuals with relapsed or refractory neuroblastoma. In a study, 12 patients received increasing doses of second-generation GD2-directed CAR-T cells and escalating intensity of chemotherapy. Researchers demonstrated that prior lymphodepletion is associated with measurable peripheral blood engraftment of GD2-CAR T-cells. However, two of six patients who received ≥108/m^2^ CAR-T cells after chemotherapy suffered 2 or 3 cytokine release syndrome (CRS), and three had regression of soft tissue and bone marrow disease [35].

An approach with 4th generation GD2-specific chimeric antigen receptor (4SCAR-GD2) is likewise being tested. In one study, 34 individuals with high risk GD2-positive neuroblastoma received safety-engineered lentivector CAR containing multiple intracellular signaling domains after cyclophosphamide and fludarabine chemotherapy. No grade 3 or 4 toxicity occurred. A total of 15% of patients after a one-year-old observation showed partial response to the treatment [36]. In another completed clinical trial, three children with neuroblastoma received infusion of GD2 CAR modified Tri-virus specific cytotoxic T-cells early after hematopoietic stem cell transplantation (HSCT). None of the participants showed infusion-related toxicity within 8 weeks of monitoring. All three patients died of disease within a year [37]. A phase I/II clinical trial carried out in Italy is enrolling children with neuroblastoma to treat them with GD2-CART01 after lymphodepleting chemotherapy. In a publication indexed to this trial Nicola Tomino et al. observed that polymorphonuclear myeloid-derived suppressor cells (PMN-MDSC) inhibits the anti-tumor cytotoxicity of different generations of GD2.CAR T-cell. Hence, patients with high levels of PMN-MDSC do not respond or lose response to the treatment [38]. In the phase I clinical trial enrolled patients with neuroblastoma and osteosarcoma are treated with iC9-GD2-CAR-VZV-CTLs in combination with a varicella zoster vaccine and lymphodepleting chemotherapy. Investigators insert the GD2-CAR gene into T cells that recognize varicella zoster virus (VZV)- cells that can remain in peripheral blood for several years. Tanaka et al. observed that GD2.CAR-VZVSTs kill either VZV- or GD2-positive target cells, yet CAR-VZVSTs re-exposed to tumor cells are dysfunctional via their CAR, but retain responsiveness to their VZV-specific TCR. Therefore, the vaccination via the TCR could provide a way to reactivate those already dysfunctional by the tumor microenvironment CAR T-cells [39].

There are other ongoing clinical trials associated with GD2 CAR-T against neuroblastoma [40,41,42,43,44,45]. In contrast, there is one trial that uses NKT cells for CAR transduction [46].

#### 3.2.2. CD171/L1-CAM—The L1-Cell Adhesion Molecule (CD171)

The L1-cell adhesion molecule (CD171) is a marker usually overexpressed in metastatic neuroblastoma. It likewise participates in the regulation of tumor cell activities. Park et al. constructed a CAR including CE7, which is an antibody binding to the L1-CAM epitope, as a single-chain antibody extracellular domain (scFv). They also created a plasmid vector that drives the transcription of CE7R and the selection-suicide fusion protein HyTK. Patients were treated with increasing doses of CE7R/HyTK+ CD8+ cytolytic T-lymphocyte (CTL). The off-tumor on-target toxicity did not appear. From the six individuals who received the infusions, only one patient experienced prolonged survival [47].

There is one ongoing phase I clinical trial that enrolled people with recurrent or refractory neuroblastoma. The study consists of three arms. One group receives second generation CD171 specific CAR T cells expressing EGFRt, another group derives third generation CD171 specific CAR T cells expressing EGFRt and the last one receives long spacer second generation CD171 specific CAR T cells expressing EGFRt. The aim of the study is to measure the dose limiting toxicity and the tumor response [48].

Lack of persistence seems to be a major hurdle in efficacy of adoptive CAR T-cell therapy. Thus, novel approaches and modifications are still required.

### 3.3. Wilms Tumor

Wilms tumor (WT), also referred as nephroblastoma, is the most common type of renal cancer that occurs in children [49]. Within the context of CAR-T cell therapy, three immunological targets are considered: glypican 3 (GPC3), epidermal growth factor receptor (EGFR) and B7-H3 (CD276).

Glypican 3 (GPC3) is a heparan sulfate proteoglycan anchored to the cell surface by a lipid molecule. It was shown that GPC3 promotes the growth of hepatocellular carcinomas (HCCs) by stimulating Wnt signaling [50]. Several studies have proven significant expression of GPC3 not only in hepatoblastoma but also in WT [51,52]. For this reason, pediatric patients diagnosed with WT are recruited for a phase-I clinical trial using GPC-3 CAR-T cells combined with IL-15 gene in the treatment of GPC3+ solid tumors [53].

Epidermal growth factor receptor (EGFR) is considered to be another target of CAR-T cells therapy as a protein that plays a role in the growth and division of certain cells by binding to a signaling molecule called epidermal growth factor (EGF) [54]. A phase-I clinical trial is the study evaluating EGFR806 CAR-T cells as a treatment of recurrent/refractory pediatric solid tumors, including WT, with two arms receiving different versions of the treatment [55].

B7-H3, as a transmembrane protein of B7 superfamily molecules [56], is a third proposed target for CAR-T cell therapy of WT. Majzner et.al found that 325 (84%) among 388 pediatric solid tumors specimens were positive for B7-H3, with 70% demonstrating high intensity staining of 2+ or 3+. All the 12 samples of Wilms tumor showed high intensity staining of 2+ or 3+. Majzner’s team constructed a second generation of B7-H3 CAR-T cells as a promising treatment option for children and young adults with recurrent or refractory solid tumors [20], which is currently being tested in a phase-I clinical trial [57].

### 3.4. Osteosarcoma

Osteosarcoma (OS), which is also referred osteogenic sarcoma, is one of the most common primary malignancies of the bone among children and adolescents [58]. The effectiveness of CAR-T cell therapy depends on accurately identifying and targeting the tumor cells. Nowadays, the most common targets in CAR-cell treatment for OS are: HER-2, GD2 [59], B7-H3 [20], IL-11 Ra [60], IGF1R [61], EphA2 [62].

#### 3.4.1. HER-2, GD2, B7-H3—Preclinical Researches and Clinical Trials

Despite the low HER-2 expression in OS [63], Ahmed et al. found that HER2-targeted CAR-T cells were able to induce immunological response in OS cells with low HER-2 expression in vitro. Mouse models with OS cells xenograft showed regression of tumor and lung metastasis after implementation of CAR-T cells [64]. Other research showed that while HER2-specific T cells were able to produce some level of tumor regression and remission in mouse models with OS lung metastasis, they did not completely eliminate the cancer cells. However, the study found that HER2-specific T cells affected the OS tumor-initiating cell (TIC) compartment in established bone tumors, impairing the self-renewal capacity of OS cells within the tumors [65]. Ahmed et al. conducted a phase I/II clinical trial among 17 patients with recurrent/refractory sarcomas, mainly OS. Patients were given escalating doses of CAR-T cells with HER-2 specific binding from 1 × 10^4^ cells/m^2^ to 1 × 10^8^ cells/m^2^. The highest dose was well tolerated and in four patients the disease became non-progressive within 3 to 14 months. Furthermore, removal of remnant matases allowed to obtain remission of three patients at 6, 12, and 16 months. Researchers have proven that HER-2 CAR-T cells can penetrate to the tumor cells and be maintained there for 6 weeks [66].

The study of Roth et al. has shown that expression of disialoganglioside (GD2) in OS is higher than in neuroblastoma [67]. Long et.al proved that all of the OS samples used in their experiment were GD2-positive. They constructed a third generation of CAR-T cells with CD28 and OX40 costimulatory domains which recognized and destroyed OS GD2+ cell lines in vitro. However, treatment was ineffective in vivo in the OS xenograft model due to accumulation of myeloid-derived suppressor cells (MDSCs). Those cells can be suppressed by all-trans retinoic acid (ATRA). For these reasons, researchers added ATRA to the therapy, which improved the effectiveness of 2SCAR-GD2-CAR-T cells in vivo [68]. Chulanetra et al. demonstrated that 4th generation of CAR-T cells may effectively destroy OS cells with GD2-expression level exceeding 80%. However, they observed that interaction between CAR-T and OS cells may induce overexpression of PD-L1 in OS cells and higher levels of PD-1 in CAR-T cells resulting in CAR-T apoptosis. The 4th generation of CAR-T cells combined with low doses of doxorubicin allowed a reduction in the expression of PD-L1 on tumor cells, which significantly improved the effectiveness of therapy [69].

An outgoing phase-I clinical trial uses third generation of CAR-T cells with safety switch vector which can induce death of the CAR-T molecules if they were to induce indesirable toxicity [42]. Another phase-I clinical trial is recruiting patients over 18 months of age diagnosed with OS or neuroblastoma. They are using GD-2 CAR-T cells combined with gene of IL-15 and caspase 9 (iC9). The IL-15 gene was included to increase the effectiveness of the GD2-CAR-T cells in attacking tumor cells whereas the iC9 gene was added as a “stop switch” that can be used to deactivate the GD2-CAR-T cells if any serious side effects occur. CAR-T cells are transfused to patients after prior lymphodepletion with cyclophosphamide and fludarabine [41]. A similar phase-I clinical trial using Cy/Flu and GD-2 CAR-T cells is also underway in patients with refractory/recurrent OS or neuroblastoma [40].

Results of Majzner et al. experiment showed very high in vivo anti-tumor activity of B7-H3 CAR-T cells based on MGA271. However, the success of the treatment may depend on the target antigen density on the surface of the tumor tissue, with higher densities potentially leading to a more effective outcome [20]. Talbot et al. used an orthotropic implantation technique in mouse model to show the effectiveness of B7-H3-targeted CAR-T cells in the treatment of primary and metastatic OS. They found that it was successful in a dose-dependent manner, meaning that higher doses of the CAR-T cells were associated with more favorable outcomes. Moreover, different doses create different forms of primary tumor response and further development of metastases. These patterns resembled the clinical surveillance of human patients and their outcomes [70]. There is a phase-I trial which is recruiting patients under 21-year-old with B7-H3-postive tumors, including OS for the purpose of establishing maximum tolerated dose (MTD) of B7-H3-CAR T cells after prior lymphodepletion [71]. Another clinical trial investigating the efficacy of CAR-T cells in treatment of B7-H3+ tumors in patients at the age of 1 to 75 years is in its early phase-I and has not recruited participants yet [72].

#### 3.4.2. IL-11Ra, IGF1R, ROR1, and EphA2—Preclinical Researches

One study conducted by Huang and colleagues showed that IL-11Ra is present in both OS cell lines and OS lung metastases. The researchers also developed IL-11Ra-targeted CAR-T cells, which were able to effectively eliminate OS cells in vitro and accumulate in lung metastases rather than in healthy surrounding lung tissue. However, not all OS cells fully express IL-11Ra so combining this therapy with other treatments may help to improve its overall efficacy and provide a more comprehensive approach to treating the cancer [63].

It was found that CAR-T cells targeting either IGF1R or ROR1 had activity against OS cells both in vivo and in vitro. These lymphocytes indicate specific toxicity against sarcoma cells in a form of IFN-γ, TNF-α, and IL-13 cytokines production in vitro. Furthermore, the scientists discovered that IGF1R or ROR1 CAR-T cells were highly effective at inhibiting the growth of sarcoma in localized and disseminated sarcoma xenograft models which resulted in a prolonged survival rate [73].

The results of Hsu’s study suggest that EphA2-directed CAR-T cells can be an effective treatment for EphA2-expressing tumors. They found that this treatment significantly reduced or eliminated tumor burden in a mouse model both in localized tumors and in tumors with disseminated metastases. However, the researchers also observed that treatment with EphA2-targeted CAR T cells may lead to an increase in EphA2-negative tumor cells, which could potentially result in tumor immune escape [62].

There is an outgoing phase-I clinical trial “Feasibility and Safety Study of Fluorescein-Specific (FITC-E2) CAR T Cells in Combination with Parenterally Administered Folate-Fluorescein (UB-TT170) For Osteogenic Sarcoma”. They are recruiting patients with refractory or recurrent/progressive osteosarcoma that has failed first line therapy for OS per NCCN or upfront Children’s Oncology Group clinical trial and do not qualify for surgical resection. The idea of the study is to transfuse autologous CD4+ and CD8+ T cells that have been genetically modified to express antiFL (anti-Fluorescin CAR-T cells) which can effectively kill tumor cells by marking them with ubiquitin (UB_TT170) beforehand. No results have been posted yet [74].

A phase-II clinical trial is recruiting patients ≥6 months and ≤80 years of age at the time of enrollment. This clinical trial aims to investigate the use of CAR-T cells in combination with low-dose chemotherapy and maintenance sarcoma vaccines as a complex treatment for sarcoma that has relapsed or progressed to a late stage [75].

### 3.5. Rhabdomyosarcoma

For children and adolescents with rhabdomyosarcoma (RMS), the most common type of malignant soft-tissue tumor, there is a lack of effective treatment options for those with metastatic disease that is resistant to standard chemotherapy [76]. Therefore, new therapeutic approaches such as CAR-T cells are required.

A case of a 2-year-old male patient from Beijing Children’s Hospital demonstrated the use of CAR-T cell therapy in the treatment of RMS. The boy was diagnosed with middle-risk embryonal RMS (IIa, TNM stage 2) of the bladder cavity, without distant metastases. Patient was subjected to 3 operations, 25 cycles of chemotherapy and 1 cycle of radiotherapy but despite this, the disease relapsed twice during the treatment. Due to the recurrent nature of the tumor it was decided to implement 4th generation of CAR-T cells therapy. Immunohistochemically results confirmed CD56+ phenotype of tumor’s tissue, which is frequently appearing marker of RMS [77,78,79]. The transfer of 4SCAR-CD56-CAR-T cells was subsequently made after the prior chemotherapy. The procedure went without any complications. The patient has been under regular observation for 3, 5 years and finally achieved complete remission with no evidence of metastases or thickening of the bladder wall [80].

Another study presented a case of a 7-year old boy with refractory HER-2+ alveolar RMS in the right calf muscle, which had metastasized to bone marrow. Despite receiving intensive chemotherapy, the child was unable to achieve remission, therefore he was qualified for a phase-I clinical trial. This clinical trial is focused on improving the effectiveness of CAR-T HER-2 therapy after lymphodepletion in patients diagnosed with advanced sarcoma. Patient’s CD8+ lymphocytes were modified to obtain HER-2 specific binding. In order to increase the stability of CAR-T cells, a CD28 co-stimulatory endo-domain was added. After three cycles of HER2-CAR-T cells following lymphodepleting chemotherapy, the patient achieved remission, which was consolidated with four more infusions without lymphodepletion. However, the disease relapsed in the bone marrow six months after the end of the therapy. The patient achieved a second remission after one cycle of lymphodepletion and HER2 CAR-T cells, followed by infusions of pembrolizumab to improve their efficacy. At the time the article was published, the patient had been in remission for 20 months [81].

### 3.6. Retinoblastoma

Retinoblastoma is the most common childhood ocular carcinoma caused by RB1 suppressor gene mutation. It can be classified in two groups: unilateral and bilateral and constitutes for 2% of all pediatric cancers [82]. Preclinical data indicates both CD171 and GD2 are effective targets on human retinoblastoma cell lines and CAR-T cell therapy is highly effective against retinoblastoma in vitro. CD171-specific CAR-T cells induce escape mechanisms, therefore targeting of two different antigens by sequential CAR-T cell applications enhances tumor cell killing [83]. Subsequent preclinical works reported about GD2-specific second-generation CAR-Ts releasing IL-15 in injectable hydrogel encapsulated form which has been delivered to mice. Not only did it eradicate RB cells but also helped to preserve mouse vision [84]. The recent data confirmed the GD2 as highly specifically targeting RB cancer cells by utilizing fourth-generation GD2-CAR T. Although, as shown by the results, the multiple stimulation by 4SCAR-GD2 T cells contributes to antigen escape. The solution may be the combination of CAR T cells with immune checkpoint inhibitors [85].

Currently, there are two phase-I clinical trials being conducted, which are recruiting children and young adults sequentially under 26 and 30 years old with, among others, retinoblastoma. The first assesses the safety, tolerability, toxicity, and feasibility of B7H3-specific CAR T infusion and CAR T cells directed at B7H3 and CD19 [57]. The latter estimates the safety, toxicity, maximum tolerated dose, and dose limiting toxicities of EGFR-specific CAR T and EGFR and CD19 cells infusion [55].

### 3.7. Ewing Sarcoma

Limited treatment efficacy of metastatic and recurrent Ewing’s sarcoma led to search for new, more effective therapies, which will be likewise highly specific to tumor lesions reducing damage to normal tissues, commonly known as a targeted therapy. CAR T-cell therapy meets given criteria and is used in research, which is focused on finding the ES tumor- specific antigens. Targets presently identified in ES comprise the vascular endothelial growth factor receptor 2 (VEGFR2), type I insulin-like growth factor receptor (IGF1R), receptor tyrosine kinase-like orphan receptor 1 (ROR1), ganglioside2 (GD2), B7-H3 (CD276), and hepatocellular receptor tyrosine kinase class A2 (EphA2) [86].

#### 3.7.1. VEGFR2, IGF1R, ROR1 and EphA2—Only Preclinical Research

Vascular endothelial growth factor (VEGF) is an endothelial cell-specific mitogen with the ability to induce physiological and pathological angiogenesis. VEGF-mediated signaling occurs in tumor cells and is an important factor of tumorigenesis. VEGFR2, which belongs to the family of VEGF receptors, is the predominant receptor tyrosine kinase (RTK), that mediates VEGF signaling in endothelial cells and drives VEGF-mediated angiogenesis [87]. Some studies have revealed the importance of VEGF in Ewing Sarcoma [88,89]. Alexander Englisch et al. made second generation CAR constructs targeting human and murine VEGFR2 to enable preclinical studies in EWS xenograft models. Biopsies were isolated from patients with EwS treated at University Children’s Hospital, Muenster. Research has shown that VEGFR2-specific CAR T cells efficiently and specifically lysed VEGFR2-expressing target cells of the corresponding species and responded to target interaction with robust antigen-specific degranulation responses and cytokine secretion. Therefore, considering the fact that human EwS are strongly vascularized and express VEGFR2 on tumor endothelial cells, it could be appropriate target for CAR T-cell therapy in ES [90].

Type I Insulin-Like Growth Factor Receptor (IGF1R) is a transmembrane receptor tyrosine kinase and is necessary for the transforming ability of several oncogenes. IGF1R has been demonstrated to have significant association with adverse outcome, shorter overall survival, and was nearly significantly associated with advanced ES tumors. Overexpression of tyrosine kinase-like orphan receptor 1 (ROR1) is supposed to be involved in ES tumor cell migration and invasiveness [73,91]. There are no clinical studies on IGF1R and ROR1 CAR T-cell models. Xin Huang et al. presented that CAR-T cells targeting IGF1R and ROR1 from healthy donors and sarcoma patients exhibit specific cytotoxicity in vitro. In the phase II study of ES, researchers found that patients treated with a fully human anti-IGF1R antibody had adverse events such as thrombocytopenia, neutropenia, and leukopenia [73].

Erythropoietin-producing Hepatocellular receptor tyrosine kinase class A2 (EphA2) is an antigen expressed in a variety of sarcomas, including ES. Kenneth Hsu et al. demonstrated that EphA2 CAR T cells have strong anti-tumor efficacy in vitro and can eliminate established ES tumors in vivo. No clinical trials associated with this receptor have started [62].

#### 3.7.2. GD2 and B7-H3—Clinical Trials Started

The disialoganglioside GD2 is a surface molecule highly expressed in ES tumor cells with restricted and low expression levels in normal tissues [92]. There are two ongoing clinical trials associated with GD2 CAR T-cell in treatment of Ewing Sarcoma. In one phase I study, patients with relapsed or refractory neuroblastoma and other GD2-positive cancers are recruited, including recurrent Ewing Sarcoma. Researchers add a new C7R gene responsible for constant cytokine supply to GD2 T-cell in order to extend T cells lifespan. The aim of this study is to find the maximum safe dose of GD2-C7R CART cells and to assess how long they can be detected in the blood and how it effects on cancer [43]. There is another phase II study on patients with late staged and/or recurrent sarcoma with poor prognosis despite complex multimodal therapy, including GD2-positive sarcomas [93].

B7-H3 tends to be highly expressed by solid tumor cells, especially during pathological angiogenesis [86]. There is a phase I, open- label, non-randomized study enrolling pediatric and young adult participants (0–26 years old) with recurrent or refractory non-CNS solid tumors. There are two groups of participants. One group will receive B7H3-specific CAR T cells only. Another group will receive CAR T cells directed at B7H3 and CD19, a B lymphocyte marker, in order to promote the expansion and persistence of the CAR T cells [57]. Another ongoing phase I trial for ≤21 years old patients with relapsed or refractory B7-H3- positive solid tumors aims to evaluate the safety and maximum tolerated dose of B7-H3-CAR T cells [71].

### 3.8. The Summary of Clinical Studies on Solid Tumors in Children

The available studies on CAR-T efficacy in pediatric solid tumors are summarized in Table 1. Therefore, all ongoing, recruiting clinical trials associated with CAR-T cell therapy in pediatric solid tumors are included in Table 2.

## 4. Limitations of Car-T Therapy

Compared with hematological malignancies, CAR therapies for pediatric solid tumors have been lagging. Due to rapid development of this method, scientists face important challenges to be solved. Describing the pediatric solid tumors, it is necessary to briefly explain main restrictions of CAR-T cell therapy hindering its treatment including antigen escape and immunosuppressive tumor microenvironment, CAR-T cell trafficking and tumor infiltration, on-target off-tumor effects and CAR-T cell-associated toxicities [100]. They are specified in Table 3 with the examples of suggested solutions. Despite the fact that research to date mainly concern adults, hematological malignancies, and preclinical data, they provide an indication for subsequent exploration of this field.

### 4.1. Antigen Escape

The first barrier of CAR immunotherapy prosperity is antigen escape when the tumor cells avoid killing by expressing alternative forms of target antigens that lack extracellular epitopes recognized by CAR-T cells. The solid tumors exhibit greater antigen heterogeneity and express antigen on a lower level than hematological malignancies [101]. The solution for this is a concomitant multiple targeting instead of focusing on one specific antigen. Such a conclusion was drawn by scientists who created tandemic CARs focused both on HER2 and IL13Rα2. They showed that a single bispecific chimeric antigen receptor molecule can be targeted in two tumor-associated antigens and significantly improve tumor control in GBM [102]. The example of bispecific therapy among children is a phase I clinical trial conducted on patients suffering from GBM [103]. To combat heterogeneity, Choe et al. Created the EGFRvIII synNotch–α-EphA2/IL13Rα2 CAR T cells which firstly target into specific but heterogenous antigen EGFRvIII and then into non-specific and homogenous EphA2 and IL13Rα2. They proved that preclinically combinatorics models exceed traditional CARs [104]. Bielamowicz et al. proposed a model of universal trivalent uCART targeting in HER2, IL13Rα2, and EphA2 in patient’s derived GBM cell lines. In this cohort study almost all tumour cells have been captured by overcoming interpatient antigen variability which increases the spectrum of possibilities [105]. There are no studies on cells obtained from pediatric GBMs and other solid tumors so it is important to verify whether these results can be useful in children.

### 4.2. Immunosuppressive Tumor Microenvironment (TME)

Tumor microenvironment (TME) consists of physical stroma barriers and immunosuppressive cells with cytokines. Therefore, the extracellular matrix and regulatory T cells (Tregs), myeloid-derived suppressor cells (MDSCs), and M2 macrophages contained in them, impair the efficacy of CAR-T cell infusion. Moreover, the immune checkpoints such as CTLA4 and PD1 as well as lymphocyte activating antigen-3 (LAG-3), T-cell immunoglobulin and mucin-domain containing-3 (TIM-3) and T cell immune-receptor with Ig and ITIM domains (TIGIT), B- and T-lymphocyte attenuator (BTLA), B7-H3, V-domain Ig suppressor of T cell activation (VISTA) and indoleamine dioxygenase (IDO) weaken the immune response [106,107].

The strategy to combat immunosuppressive TME is the development of immune-checkpoint inhibitors (ICI) targeted into these immune checkpoint molecules and ligands, immune suppressor cells, targeting suppressive cytokines and molecules in the tumor microenvironment and the production of proinflammatory cytokines [108]. In other words, the use of ICI enables the strengthening of tumor responses while weakening the immunosuppressive mechanisms. It is suggested that the combination with other ICI, radiotherapy, chemotherapy, or targeted agents may be more effective in the future [109].

Another promising option is the oncolytic virus infection which enhances the expression of programmed cell death 1 (PD1) ligand 1 (PDL1) or increases the recruitment and activation of local T cells. But further research on combination CAR-T with oncolytic viruses are necessary [110]. There are preclinical studies which suggest that oncolytic viruses deserve more investigations in children with DIPG [111]. The conducted study revealed that HSV-1 significantly increased the number of tumor-infiltrating lymphocytes in pediatric patients with HGG [112]. The viruses can be applied in vaccines in another combinatorial method with checkpoint inhibitors [113]. Unfortunately, there are still not enough studies to overcome TME in pediatric solid tumors. For instance, the pediatric GBM may have less immunosuppressive strategies compared with adults, so the data concerning therapy dedicated to pediatric patients needs to be investigated [114].

### 4.3. Restricted Trafficking and Limited Tumor Infiltration

As described earlier, TME and physical tumor barrier limit the capacity of CAR-T cells to traffic and infiltrate the solid tumors and to exert the cytotoxic effect. The solution to overcome this hurdle is local intrathecal or intraventricular delivering of CAR-T cells as it was previously described in solid tumors [14,22,25,26]. According to aforementioned sources, locoregional delivery is associated with less systemic toxicity such as CRS, attenuated on target off-tumour effects, decreased locally immunosuppression while enhanced levels of proinflammatory cytokines and exposition of CARs to cancer cells. Furthermore, an alternative solution is direct migratory engineering of T Cells. It is based on ectopic chemokine receptor expression (e.g., CCR4), degradation of the extracellular matrix (by ectopic expression of heparanase or hyaluronidases) or alter chemokine expression (recruitment of subsequent T cells) [115].

### 4.4. On-Target Off-Tumor Effects (OTOT)

The problem of OTOT concerns mainly solid tumors and its antigens in healthy tissues since hematological malignancies are usually restricted to cells of hematological lineage. OTOT is based on recognition and distraction of nonmalignant tissues, expressing targeted antigen. The identification of this antigen leads to destroying tissues by effector functions such as: releasing of perforin and granzymes, upregulation of T-cell surface molecules to induce target cell apoptosis or secretion of cytokines including IFNγ and/or TNF. There are several examples which present OTOT as the cause of severe undesirable effects. The solution for this may be modification of the scFv and other domains of the CAR or logic-gated CAR T cells. Another possible approach is the targeting of tumor-restricted post-translational modifications such as solid tumor overexpressed truncated O-glycans [116,117]. Yiqian Wu et al. suggested the use of focused ultrasounds (FUS) to activate and control functions of CAR-T cells by MRI-guided at local tumor sites and proved this technique decreases on-target off-tumor toxicity of CAR T cell therapy [118].

### 4.5. Toxicity of the CAR-T Cell Therapy

The last obstacle is the toxicity of CAR-T therapy, particularly CRS and neurotoxicity. Both may not occur simultaneously and the CRS recovery does not exclude the neurotoxicity. Depending on the severity of these conditions they may resolve or endanger life. Risk increases with the higher tumor mass and dose of CAR-T cells. These inflammatory toxicities are mitigated by corticosteroids and cytokine inhibitors such as tocilizumab (IL-6 inhibitor). Tocilizumab is more effective in CRS treatment whereas corticosteroids are a first-line agent to combat severe neurologic toxicity and for those with CRS who do not respond to tocilizumab [119,120]. However, because of the limitations of this method, there are other antibodies such as siltuksymab which require further studies [121]. Another inhibitor—anakinra (IL-1 inhibitor) proved to be effective both in CRS and neurotoxicity in preclinical studies [122].

**Table 3:** The summary of main obstacles associated with targeting CAR-T cells into solid tumors and the main ways to handle them.

Limitations to Overcome	Main Potential Solution	References
Antigen escape	Multiple target therapy	[102,104,105]
Immunosuppressive TME	Usage of immune checkpoint inhibitors and oncolytic viruses	[108,109,110,111,112]
Restricted trafficking and limited infiltration	Local delivery of CAR-T	[14,22,25]
On-target off-tumor effects	Modification of the scFv and other domains	[117]
Toxicity of the therapy	Administration of corticosteroids and cytokine inhibitors	[120]

TME—tumor microenvironment; CAR-T—chimeric antigen receptor T cells

## 5. Conclusions

CAR-T cell therapy has shown promising results in the treatment of pediatric solid tumors, but further research is needed to fully understand its safety and efficacy. Studies have demonstrated that CAR-T cells can target and destroy cancer cells in pediatric solid tumors, leading to significant tumor regression and improved survival rates. The diversity of factors affecting the success of CAR-T cells has great importance in developing new methods in order to target them precisely to the surface of solid tumors. Given the fact that the encountered difficulties affect each other and may occur at the same time, the implementation of as many synergistic actions as possible is desirable. While there have been some successful cases, the treatment is still considered experimental and many studies are currently being conducted to confirm its effectiveness in larger populations. The results of the clinical trials, which are presently in an early stage, should be expected, which will allow us to refine the therapy and find out its long-term effects. Although the CAR-T cell therapy is still considered investigational, preliminary results have been encouraging.

## Figures and Tables

**Table 1:** Available studies on CAR-T efficacy in pediatric solid tumors.

Tumor	Number of Patients Treated with CAR-T	Treatment Effects	References
Diffuse Intrinsic Pontine Glioma	3	6 months after treatment: 2 pts- increased tumor bulk with infiltration in the right brachium pontis and dentate nuclei 1 pt- mild decrease in tumor size	[22]
H3K27M-mutated diffuse midline gliomas	4	3 pts- initially: exhibited clinical and radiographic improvement, 2 pts died in 7 and 10 months after first cell infusion, 1 pt survived after the data cutt-off 1 pt- death in 3 months after first cell infusion	[25]
Neuroblastoma	11	6 weeks after treatment: 4 pts- no evidence of disease 2 pts- stable disease 2 pts- tumor necrosis 2 pts- progressive disease 1 pt- partial response	[32]
Neuroblastoma	19	6 weeks after treatment: 8 pts- no evidence of disease 4 pts- progressive disease 2 pts- complete response 2 pts- tumor necrosis 2 pts- stable disease 1 pt- partial response	[33]
Neuroblastoma	11	6 weeks after treatment: 6 pts- progressive disease 5 pts- stable disease	[34]
Neuroblastoma	12	6 pts- no in vivo expansion of CAR T-cells was detected, no immune activation or antitumor activity was seen 3 pts- disease progression 2 pts- mixed response 1 pt- near complete clearance bone marrow infiltration	[35]
Neuroblastoma	10	6 months after treatment: 6 pts- stable disease 4 pts- progressive disease	[36]
Neuroblastoma	3	All died of the disease within a year	[37]
Neuroblastoma	6	56 days after treatment: 5 pts- progressive disease 1 pt- partial response	[47]
Osteosarcoma (16 pts) Ewing sarcoma (1 pt) Primitive neuroectodermal tumor (1 pt) Desmoplastic small round cell tumor (1 pt)	19	6 weeks after treatment: 13 pts- progressive disease 4 pts- stable disease 2 pts- not evaluable	[66]
Rhabdomyosarcoma	1	Complete remission	[80]
Rhabdomyosarcoma	1	In remission for 20 month at the time of the report	[81]

**Table 2:** All ongoing, recruiting clinical trials connected with CAR-T cell therapy in pediatric solid tumors.

Solid Tumor	Receptor	Phase of Research	Responsible Party	References
Brain Tumors	GD2	Phase 1	Franco Locatelli, Bambino Gesù Hospital and Research Institute	[29]
		Phase 1	Bilal Omer, Baylor College of Medicine	[94]
		Phase 1	Crystal Mackall, MD, Stanford University	[95]
	HER2	Phase 1	Nabil Ahmed, Baylor College of Medicine	[96]
		Phase 1	Rebecca Gardner, Seattle Children’s Hospital	[97]
	B7H3	Phase 1	Rebecca Gardner, Seattle Children’s Hospital	[98]
Neuroblastoma	GD2, PSMA and CD276	Phase 2	Shenzhen Geno-Immune Medical Institute	[45]
Osteosarcoma	CD276	Early Phase 1	PersonGen BioTherapeutics (Suzhou) Co., Ltd.	[72]
	FITC-E2	Phase 1	Rebecca Gardner, Seattle Children’s Hospital	[74]
Neuroblastoma Osteosarcoma	GD2	Phase 1	National Cancer Institute (NCI)	[40]
		Phase 1	UNC Lineberger Comprehensive Cancer Center	[41]
Neuroblastoma Osteosarcoma Ewing Sarcoma Rhabdomyosarcoma	GD2	Phase 1	Bilal Omer, Baylor College of Medicine	[43]
Neuroblastoma Osteosarcoma Ewing Sarcoma		Phase 2	Franco Locatelli, Bambino Gesù Hospital and Research Institute	[99]
Neuroblastoma Osteosarcoma Rhabdomyosarcoma Ewing Sarcoma Wilms Tumor	B7H3	Phase 1	Rebecca Gardner, Seattle Children’s Hospital	[57]
		Phase 1	St. Jude Children’s Research Hospital	[71]
	EGFR806	Phase 1	Rebecca Gardner, Seattle Children’s Hospital	[55]
Rhabdomyosarcoma Wilms Tumor	GPC3	Phase 1	Andras Heczey, Baylor College of Medicine	[53]
Osteosarcoma Ewing Sarcoma	GD2, PSMA, Her2, CD276 and other markers	Phase 2	Shenzhen Geno-Immune Medical Institute	[75]
	CD133, GD2, Muc1, CD117 and other markers	Phase 2	Lung-Ji Chang, Shenzhen Geno-Immune Medical Institute	[93]

## Data Availability

Not applicable.
